# Relationship Between Leaving Children at Home Alone and Their Mental Health: Results From the A-CHILD Study in Japan

**DOI:** 10.3389/fpsyt.2018.00192

**Published:** 2018-05-25

**Authors:** Satomi Doi, Takeo Fujiwara, Aya Isumi, Manami Ochi, Tsuguhiko Kato

**Affiliations:** ^1^Department of Global Health Promotion, Tokyo Medical and Dental University (TMDU), Tokyo, Japan; ^2^Japan Support Center for Suicide Countermeasures, National Center of Neurology and Psychiatry, Tokyo, Japan; ^3^Department of Social Medicine, National Research Institute for Child Health and Development, Tokyo, Japan

**Keywords:** leaving children at home alone, resilience, difficult behavior, prosocial behavior, Japan

## Abstract

Leaving children at home alone is considered a form of “neglect” in most developed countries. In Japan, this practice is not prohibited, probably because this country is considered to have relatively safe communities for children. The impact of leaving children at home alone on their mental health is a controversial issue, and few studies have examined it to date. The aim of this study was to examine the impact of leaving children aged 6 or 7 years at home alone on their mental health, focusing on both the positive and negative aspects; that is, resilience, difficult behavior, and prosocial behavior. Data from the Adachi Child Health Impact of Living Difficulty (A-CHILD) study were used. The caregivers of all children in the first grade in Adachi City, Tokyo, were targeted, of whom 80% completed the questionnaire (*n* = 4,291). Among the analytical sample which comprises those who completed both exposure and outcome variables (*n* = 4,195), 2,190 (52.2%) children had never been left at home alone, 1,581 (37.7%) children were left at home alone less than once a week, and 424 (10.1%) children were left at home alone once a week or more. Child resilience was measured using the Children's Resilient Coping Scale, and difficult behavior (emotional symptoms, conduct problems, hyperactivity/inattention, and peer relationship problems) and prosocial behavior using the Strength and Difficulty Questionnaire. Multivariate regression analyses were performed to examine the dose-response association between leaving children at home alone and child mental health, followed by propensity-score matching as a pseudo-randomized controlled trial to reduce potential confounding. The results showed that leaving children at home alone once a week or more, but not less than once a week, was associated with total difficulties scores, especially conduct problems, hyperactivity/inattention, and peer relationship problems. These findings indicate that leaving children at home alone should be avoided in Japan, as is recommended in North America.

## Introduction

Leaving children at home alone is considered a form of “neglect” in most developed countries (1). In the United States, some states have laws that relate to the minimum age of children who can be left at home alone in order to protect them from neglect, accidents, and crime, or they provide guidelines that can assist parents in their decision on leaving their children at home alone (2, 3). For example, the Illinois law stipulates that children aged less than 14 years old should not be left at home alone (4). In Maryland, the law permits parents to leave children aged 8 years or above alone^1^.

However, the impact of leaving children at home alone is controversial. If children are mature enough to stay safely at home, being alone might provide them with unique opportunities to enhance their independence, responsibility, and confidence (5, 6). Moreover, older adolescents are more likely to report positive responses to the question on being at home alone, such as having time to read a book and do homework without distraction, and having the opportunity to invite friends over or to go out with friends (7).

On the other hand, the majority of the preceding studies on this topic report negative responses among adolescents. Many studies in North America have shown that adolescents left at home alone feel lonely, have worries, and experience fear, and are also at risk of antisocial behavior such as truancy, stealing, and drinking (6–8). Mertens et al. (9) also showed that middle-grade students, aged 12–14 years in the United States who were left at home alone for 3 h or more tended to show higher levels of depression, behavior problems, low self-esteem, and low academic efficacy.

Surprisingly, few studies have examined the impact of leaving preadolescent children at home alone (10, 11). A low prevalence of young children being left at home alone in developed countries has been reported (12), possibly because of the law or guidelines that prohibit young children being left at home alone. In their study of 206 children in the first to fourth grades in the United States, Marshall et al. (11) indicated that leaving children at home alone was associated with behavioral problems, especially in low-income families. However, the sample size of that study was small, and the impact of leaving young children at home alone on their development needs to be investigated in a study with a larger sample size.

In contrast to North America, Japan has no law or guidelines regarding leaving children at home alone, including supervision by older siblings, probably because leaving children at home alone may not be the Japanese norm. In comparison to the Western culture, the Japanese parenting style might be more likely to be interdependent, rather than independent [e.g., (13)], which promotes the development of autonomy (14). This assumption is supported by the finding that about 95% of children in the first and second grades always have a dinner with parents that is, most children are not left alone until dinner time (15).

Another possible reason for this lack of law or guidelines is that community living offers a relatively safer neighborhood for children in Japan (16), as shown by its lowest homicide rate among Organization for Economic Co-operation and Development (OECD) countries (17). A safe living environment is one of the factors that influence the parental decision to leave children at home alone (18), as revealed in an empirical study of 5- to 7-year-old children (12). In a previous study that examined Japanese parents' perception of risk to elementary school children, more than 40% of them perceived leaving a child home alone as “safe” or “slightly safe” and about 90% accepted leaving children at home alone (19). Rather than homicide rate among neighborhoods, the perception of neighborhood safety or social capital [e.g., (20)], both developed based on social trust among neighbors, may be more feasible to assess the impact of neighborhood considering the low number of homicide in Japan. To date, no previous study has revealed a direct association between the impact of social capital and leaving children at home alone.

Social capital is defined as “the resources available to individuals or groups through their social connections” (21). In Japan, which is known to have rich social capital [as reported in narrative studies; (22)], higher social capital has been found to promote better parenting (23). Further, social capital is positively associated with child mental health [e.g., (24–26)]. Therefore, social capital may confound the association between leaving children at home alone and their mental health, and thus, it is needed to elucidate the impact of leaving children at home alone on their mental health by the level of social capital. Additionally, income, siblings, maternal education, or caregiver's mental health are associated with leaving children at home alone (8, 27) and child mental health (28, 29). Therefore, we need to assess the effect of social capital, income, siblings, maternal education, and caregiver's mental health.

Our research was conducted as a large project called the Adachi Child Health Impact of Living Difficulty (A-CHILD) study, which examined the health and living environment of children in the first grade in all elementary schools (69 schools) in Adachi City, Tokyo. Adachi City, known as a deprived area in Tokyo (e.g., the unemployment rate was 7.1% in 2010 (30), which is higher than Japan's average of 3.1% in 2016 (31) is keen on tackling with child poverty, which made the current study feasible. Participants were young Japanese children, and the present study has gathered more data than that in a previous study conducted in the United States (11). In the present study, we aimed to examine the association between leaving young children aged 6 or 7 years at home alone and their mental health, focusing on both positive and negative aspects; that is, resilience, difficult behavior, and prosocial behavior, adjusting for the effects of social capital, status of household, and caregiver's mental health.

## Methods

### Participants

**Figure 1:**
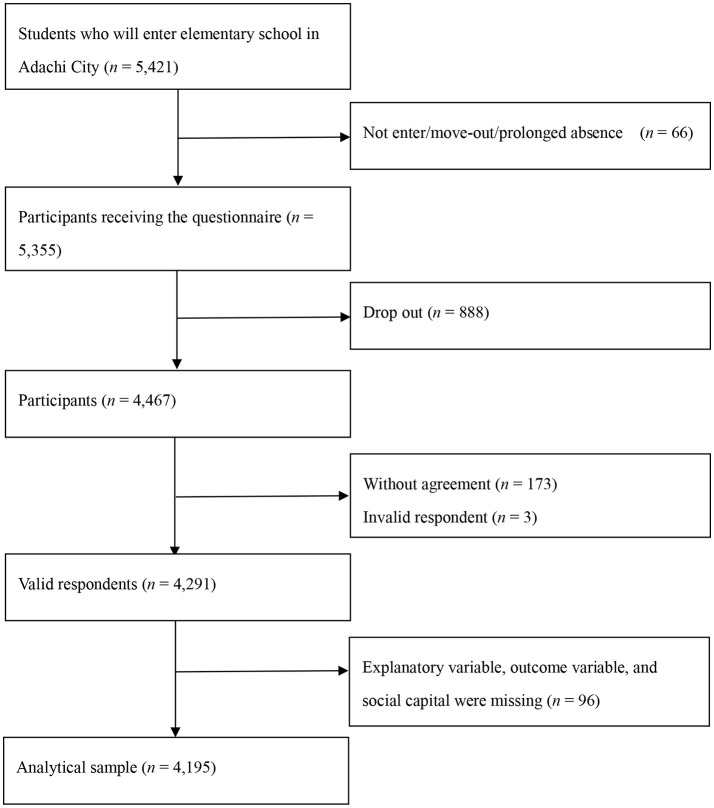
Requirement flow chart.

We used data from the A-CHILD study performed in 2015. The survey covered all 69 public elementary schools in Adachi City, Tokyo, Japan. In 2015, self-reported questionnaires with anonymous unique IDs were distributed to 5,355 children in the first grade in elementary school, aged 6–7 years. Children took the questionnaire back home, caregivers entered their responses, and children submitted the filled out response sheet to their school. A total of 4,467 participants returned the questionnaire (response rate = 83.4%). Among respondents, 4,291 participants provided informed consent (valid response rate = 80.1%). Among valid respondents, 96 participants were excluded as explanatory variable, outcome variables, and social capital were missing (Figure 1). Among analytical sample, 90.8 and 7.4% were mothers and fathers, respectively. The A-CHILD protocol was approved by the Ethics Committee in National Center for Child Health and Development (No. 1187).

### Measurements

#### Explanatory variable

##### Leaving children at home alone

The caregivers were asked how often their children stayed at home alone, more than one hour during weekday, which was rated as 1 (*never*), 2 (*one to eleven times a year*), 3 (*one to three times a month*), and 4 (*once a week or more*). In this study, 0 (*never*) was categorized as 0 (never left at home alone), 2 (*one to eleven times a year*) to 3 (*one to three times a month*) were categorized as 1 (less than once a week), and 4 (*once a week or more*) was categorized as 2 (once a week or more).

#### Outcome variables

##### Resilience

Child resilience was assessed using the Children's Resilient Coping Scale (CRCS), which has eight items developed by Japanese experts to suit the Japanese context based on previous studies related to resilience (32–35) and coping (36–38). One study showed that the CRCS has high internal consistency (Chronbach's alpha = 0.80) and sufficient validity (39). The eight items of the CRCS are 1) speaks positively about their future 2) tries to do their best 3) able to take teasing or mean comments well 4) knows how to properly greet others 5) able to get ready for school, study, and do his/her chores without directions 6) seeks appropriate advice when necessary 7) able to give up on things they want or do things that they do not like to do for better future outcomes, and 8) able to ask questions to learn about what they do not understand. For these items, respondents (i.e., caregivers) rated child resilience/coping behaviors using a scale of 0 (*never*) to 4 (*very frequently*). Total score was converted into 0–100 to help interpretation of coefficients from statistical analysis, and a higher total score indicates higher level of resilience.

##### Difficult behavior and prosocial behavior

The Japanese version of the Strength and Difficulties Questionnaire [SDQ; (40)] was translated from the English version of the SDQ (41). The SDQ, which is an others-reported tool, has 25 items and consists of five subscales: emotional symptoms, conduct problems, hyperactivity/inattention, peer relationship problems, and prosocial behavior. The total difficulties score is calculated using the sum of four subscale scores (emotional symptoms, conduct problems, hyperactivity/inattention, and peer relationship problems). Respondents rated the items on a scale of 0 (*not true*) to 2 (*certainly true*). In this study, total scores (i.e., total difficulties score, scores of emotional symptoms, conduct problems, hyperactivity/inattention, peer relationship problems, and prosocial behavior) were converted into 0–100 to help interpretation of coefficients from statistical analysis. Higher scores for total difficulties, emotional symptoms, conduct problems, hyperactivity/inattention, and peer relationship problems indicate that children have more difficulties, while a higher prosocial behavior score means more prosocial behavior. In this study, the Cronbach's alpha for total difficulties score and prosocial behavior score were 0.78 and 0.70 respectively.

### Demographic data

#### Respondents

We also assessed the respondents, regardless of whether the person was the mother, father, or any other caregiver.

#### Child characteristic

The parents or caregivers were asked about the child's sex (boy or girl).

#### Family background

The caregivers were asked about the child's siblings (younger sibling, older sibling, or no siblings), whether they were living with grandparents (yes or no), marital status (married or unmarried), mother's age (< 30, 30–34, 35–39, 40–44, or >45 years), maternal education (high school or less, some college education, college education or higher, or other/unknown), maternal occupation (full-time/part-time job, self-employed, or not working), annual household income (< 500,000, 500,000–999,999, 1,000,000–1,999,999, 2,000,000–2,999,999, 3,000,000–3,999,999, 4,000,000–4,999,999, 5,000,000–5,999,999, 6,000,000–7,499,999, 7,500,000–9,999,999, or ≥ 10,000,000 yen, or unknown).

#### Parent's or caregiver's mental health

The respondents' anxiety and mood status was assessed using the Japanese version of Kesseler 6 [K6; (42)]. Scores on this tool range from 0 to 24 and estimated cut-off points is a score of 4/5 (43). Higher scores indicate frequent problems on psychological distress. In this study, the Cronbach's alpha for the tool was 0.89.

#### Social capital

Respondents rated three items on a scale of 1 (*true*) to 5 (*false*). The items were “my community can be trusted,” “my community is cohesive,” and “neighbors in my community help each other.” These items have been used in an earlier study (23, 44). In the present study, 1 (*true*) to 2 (*somewhat true*) were categorized as 1 (high social capital), while 3 (*cannot say*) to 5 (*false*) were categorized as 0 (low social capital). The Cronbach's alpha for the tool was 0.86.

### Ethics

This study was approved by the Ethics Committee of the National Center for Child Health and Development (approval number: 1147).

### Statistical analysis

First, multivariate regression analyses were conducted to examine the impact of leaving children at home alone on child mental health, adjusting child's sex, living with an older sibling, living with a younger sibling, living with grandparents, maternal age, maternal education, marital status, maternal occupation, income, K6 score, and social capital (Model 1). Moreover, multivariate regression analyses, including interaction term (interaction between leaving children at home alone and social capital), were conducted to examine whether social capital moderates the association between leaving children at home alone and their mental health (Model 2). To deal with missing data in covariates (child's sex, maternal age, maternal education, marital status, maternal occupation, income, and K6 score), multiple imputation was performed using complete data, i.e., status of leaving children at home alone, CRCS score, SDQ total difficulties, prosocial behavior, emotional symptoms, conduct problems, hyperactivity/inattention, peer relationship problems sores, social capital status, and interaction term of leaving children at home alone and social capital (100 imputed datasets).

Second, propensity-score (PS) matching was used to examine the differences in the characteristics between the two conditions when estimating the impact of leaving children at home alone on child mental health. Thus, we conducted PS matching between children who were never left at home alone and those left at home alone less than once a week, and between children never left at home alone and those left at home alone once a week or more. The possible confounders were demographic variables such as child's sex, having a younger sibling, having an older sibling, living with grandparents, maternal age, marital status, maternal education, maternal occupation, income, K6 score, and social capital, as listed in **Table 3**. These confounders were selected based on previous studies (8, 27). In PS matching, missing data of confounders were used as dummy variable. Adjusting for these possible confounders, the PS model was estimated using a logistic regression model. PS matching used the following algorithm: 1:1 optimal match with calipers up to 0.01 and no replacement. Before and after PS matching, the balance in the possible confounders in the two conditions within the matched pairs was assessed using the standardized bias. Of the children who were left at home alone less than once a week, 76.8% (*n* = 1,214) were matched to similar children who were never left at home alone. Of the children who were left at home alone once a week or more, 89.4% (*n* = 379) were matched to similar children who were never left at home alone. Using the matched pairs, regression analyses were conducted to examine the impact of leaving children at home alone on resilience (CRCS), total difficulties, emotional symptoms, conduct problems, hyperactivity/inattention, peer relationship problems, and prosocial behavior (SDQ). The data were analyzed using STATA version 13.1.

## Results

### Children left at home alone

Table 1 shows the distribution of the CRCS, SDQ scores, and characteristics by status of leaving children at home alone. In the present study, 2,190 children (52.2%) had never been left at home alone, 1,581 children (37.7%) were left at home alone less than once a week, and 424 children (10.1%) were left at home alone once a week or more among 4,195 children in Adachi City. Children left at home alone less than once a week were less likely to have younger sibling (*p* < 0.001), more likely to have older sibling (*p* < 0.001), less likely to live with grandparents (*p* < 0.001), and their caregiver showed higher K6 score (*p* < 0.01), compared with those who had never been left at home alone. Similarly, children left home alone once a week or more less likely to have younger sibling (*p* < 0.001), more likely to have older sibling (*p* < 0.001), less likely to live with grandparents (*p* < 0.001), their caregivers were more likely to be unmarried (*p* < 0.001), their mothers were more likely working full-time or part-time (*p* < 0.001), their annual household income were less likely to be low (i.e., less than 3 million yen) (*p* = 0.06), and their caregiver showed higher K6 score (*p* < 0.01) compared with those who had never been left at home alone.

**Table 1:** Distribution of characteristics without multiple imputation.

		**Leaving children at home alone [*****n*** **(%) or mean (*****SD*****)]^a^**				***p*****-value**^a^	
		**Total**	**Never**	**<1/w**	**≥1/w**	**Never vs. <1/w**	**Never vs. ≥1/w**
		**(*n* = 4,260)**	**(*n* = 2,215)**	**(*n* = 1,611)**	**(*n* = 434)**		
CRCS	Total	66.14 (15.41)	66.64 (15.51)	64.77 (21.40)	63.77 (16.02)	0.27	<0.001
SDQ	Total difficulties score	24.83 (13.28)	24.80 (13.34)	24.28 (13.02)	27.04 (13.74)	0.24	<0.01
	Emotional symptoms	19.59 (18.71)	19.81 (18.87)	19.22 (18.46)	19.91 (18.78)	0.36	0.92
	Conduct problems	24.79 (18.52)	19.81 (18.87)	24.05 (17.70)	28.15 (19.64)	0.31	<0.001
	Hyperactivity/inattention	36.25 (23.33)	24.66 (18.82)	35.71 (22.92)	39.74 (24.34)	0.75	<0.01
	Peer relationship problems	18.78 (16.83)	35.95 (23.38)	18.24 (16.41)	20.44 (17.29)	0.27	0.08
	Prosocial behavior	66.10 (20.43)	66.27 (20.91)	66.22 (19.48)	64.77 (21.40)	0.93	0.17
Child sex	Boy	2,185 (51.3)	1,109 (50.1)	839 (52.1)	237 (54.6)	0.23	0.09
	Girl	2,070 (48.6)	1,102 (49.8)	771 (47.9)	197 (45.4)		
	Missing	5 (0.1)	4 (0.2)	1 (0.1)	0 (0)		
Younger sibling	Yes	1,823 (42.8)	1,046 (47.2)	631 (39.2)	146 (33.6)	<0.001	<0.001
	No	2,437 (57.2)	1,169 (52.8)	980 (60.8)	288 (66.4)		
Older sibling	Yes	2,022 (47.5)	687 (31.0)	1,010 (62.7)	325 (74.9)	<0.001	<0.001
	No	2,238 (52.5)	1,528 (69.0)	601 (37.3)	109 (25.1)		
Grandparents	Yes	456 (10.7)	300 (13.5)	129 (8.0)	27 (6.2)	<0.001	<0.001
	No	3,804 (89.3)	1,915 (86.5)	1,482 (92.0)	407 (93.8)		
Maternal age	<30	222 (5.2)	110 (5.0)	79 (4.9)	33 (7.6)	0.89	0.08
	30–34	762 (17.9)	397 (17.9)	287 (17.8)	78 (18.0)		
	35–39	1,451 (34.1)	754 (34.0)	552 (34.3)	145 (33.4)		
	40–44	1,305 (30.6)	688 (31.1)	503 (31.2)	114 (26.3)		
	>45	418 (9.8)	210 (9.5)	159 (9.9)	49 (11.3)		
	Missing	102 (2.4)	56 (2.5)	31 (1.9)	15 (3.5)		
Marital states	Unmarried	373 (8.8)	186 (8.4)	117 (7.3)	70 (16.1)	0.42	<0.001
	Married	3,775 (88.62)	1,971 (89.0)	1,454 (90.3)	350 (80.7)		
	Missing	112 (2.6)	58 (2.6)	40 (2.5)	14 (3.2)		
Maternal education	High school or less	1,521 (35.7)	775 (35.0)	569 (35.3)	177 (40.8)	0.21	0.05
	Some college	1,756 (41.22)	903 (40.8)	687 (42.6)	166 (38.3)		
	College or more	869 (20.4)	483 (21.8)	309 (19.2)	77 (17.7)		
	Other/unknown	114 (2.68)	54 (2.4)	46 (2.9)	14 (3.2)		
Maternal occupation	Full-time/part-time	2,412 (56.6)	1,206 (54.5)	871 (54.1)	335 (77.2)	0.15	<0.001
	Self-employed	208 (4.9)	99 (4.5)	89 (5.5)	20 (4.6)		
	Not-working/housewife	1,456 (34.2)	825 (37.3)	572 (35.5)	59 (13.6)		
	Missing	184 (4.3)	85 (3.8)	79 (4.9)	20 (4.6)		
Income	<3 million yen	488 (11.5)	1,754 (79.2)	1,280 (79.5)	320 (73.7)	0.69	0.04
	≥ 3 million yen	3,354 (78.7)	252 (11.4)	171 (10.6)	65 (15.0)		
	Missing	418 (9.8)	209 (9.4)	160 (9.9)	49 (11.3)		
K6	<5	3,001 (70.5)	1,630 (73.6)	1,117 (69.3)	254 (58.5)	0.01	<0.001
	≥5	1,211 (28.4)	560 (25.3)	477 (29.6)	174 (40.1)		
	Missing	48 (1.1)	25 (1.1)	17 (1.1)	6 (1.4)		
Social capital	Trust	2,289 (53.7)	1,185 (53.5)	880 (54.6)	224 (51.6)	0.09	0.13
	Not trust	1,928 (45.3)	1,015 (45.8)	710 (44.1)	203 (46.8)		
	Missing	43 (1.0)	15 (0.7)	21 (1.3)	7 (1.6)		
Responder	Mother	3,858 (90.6)	1,987 (89.7)	1,476 (91.6)	395 (91.0)	0.06	0.70
	Other	344 (9.0)	201 (1.1)	108 (6.7)	35 (9.1)		
	Missing	58 (1.4)	27 (1.2)	27 (1.7)	4 (0.9)		

*SD, Standard Deviation; CRCS, Children's Resilient Coping Scale; SDQ, Strength and Difficulties Questionnaire; K6, Kesseler 6*.

a *p-value for t-test or chi-squared test*.

### Multivariate regression analysis

Table 2 shows the coefficients of leaving children at home alone for CRCS and SDQ scores by multivariate regression analysis using all data. In terms of the CRCS, in crude model, leaving children at home alone once a week or more showed significantly lower resilience (β = −2.73, 95% CI = −4.34 to −1.13) compared with never leaving children at home alone, and further adjustment of potential confounders, including social capital, the association remain significant and point estimate was similar (β = −2.45, 95% CI = −4.09 to −0.80). Further, leaving children at home alone less than once a week was not significantly associated with resilience in crude model (β = −0.46, 95% CI = −1.45 to 0.54) and after adjustment of potential confounders (β = −0.91, 95% CI = −1.93 to 0.10) (Model 1). As shown in Model 2, social capital did not significantly moderate the association between leaving children at home alone and resilience (p for interaction term = 0.12), suggesting that high or low social capital did not differentiate the association between leaving children at home alone and resilience.

**Table 2:** Results of regression analyses (*n* = 4,195).

				**Crude**	**Adjusted (Model 1)^b^**	**Adjusted (Model 2)^c^**
				**β (95%CI)**	**β (95%CI)**	**β (95%CI)**
CRCS	Total score	Leaving children at home alone	Never	Ref	Ref	Ref
			<1/w	−0.46 (−1.45 to 0.54)	−0.91 (−1.93 to 0.10)	−0.34 (−1.59 to 0.91)
			≥1/w	−2.73^**^ (−4.34 to −1.13)	−2.45^**^ (−4.09 to −0.80)	−1.32 (−3.49 to 0.85)
		Social capital			3.89^**^ (2.97–4.81)	4.50^**^ (3.30–5.71)
		Interaction term^a^				−1.07 (−2.42 to 0.28)
SDQ	Total difficulties	Leaving children at home alone	Never	Ref	Ref	Ref
			<1/w	−0.52 (−1.38 to 0.34)	0.40 (−0.45 to 1.25)	−0.16 (−1.20 to 0.88)
			≥1/w	2.17^**^ (0.79–3.55)	2.22^**^ (0.85–3.59)	1.11 (−0.70 to 2.92)
		Social capital			−2.64^**^ (−3.41 to −1.87)	−2.25^**^ (−4.26 to −2.24)
		Interaction term^a^				1.05 (−0.07 to 2.18)
	Emotional symptoms	Leaving children at home alone	Never	Ref	Ref	Ref
			<1/w	−0.60 (−1.81 to 0.61)	0.12 (−1.12 to 1.37)	−0.31 (−1.84 to 1.22)
			≥1/w	0.05 (−1.89 to 1.99)	0.19 (−1.83 to 2.20)	−0.66 (−3.32 to 2.00)
		Social capital			−2.83^**^ (−3.96 to −1.70)	−3.29^**^ (−4.77 to −1.82)
		Interaction term^a^				0.81 (−0.84 to 2.46)
	Conduct problems	Leaving children at home alone	Never	Ref	Ref	Ref
			<1/w	−0.65 (−1.84 to 0.54)	−0.22 (−1.45 to 1.01)	−0.71 (−2.22 to 0.80)
			≥1/w	3.50^**^ (1.58–5.42)	2.61^*^ (0.62–4.60)	1.65 (−0.97 to 4.28)
		Social capital			−1.88^**^ (−2.99 to −0.76)	−3.40^**^ (−3.86 to −0.95)
		Interaction term^a^				0.91 (−0.72 to 2.54)
	Hyperactivity/inattention	Leaving children at home alone	Never	Ref	Ref	Ref
			<1/w	−0.23 (−1.73 to 1.28)	1.01 (−0.50 to 2.52)	0.20 (−1.66 to 2.05)
			≥1/w	3.55^**^ (1.13–5.97)	3.38^**^ (0.93–5.83)	1.79 (−1.44 to 5.02)
		Social capital			−2.71^**^ (−4.09 to −1.34)	−3.59^**^ (−5.39 to −1.79)
		Interaction term^a^				1.52 (−0.49 to 3.52)
	Peer relationship problems	Leaving children at home alone	Never	Ref	Ref	Ref
			<1/w	−0.61 (−1.70 to 0.47)	0.70 (−0.42 to 1.81)	0.17 (−1.19 to 1.54)
			≥1/w	1.58 (−0.17 to 3.33)	2.69^**^ (0.89–4.49)	1.67 (−0.70 to 4.05)
		Social capital			−3.15^**^ (−4.16 to −2.14)	−3.71^**^ (−5.03 to −2.39)
		Interaction term^a^				0.97 (−0.50 to 2.45)
	Prosocial behavior	Leaving children at home alone	Never	Ref	Ref	Ref
			<1/w	−0.03 (−1.35 to 1.29)	−0.26 (−1.63 to 1.12)	0.21 (−1.48 to 1.90)
			≥1/w	−1.41 (−3.54 to 0.71)	−1.36 (−3.59 to 0.86)	−0.45 (−3.39 to 2.49)
		Social capital			2.05^**^ (0.80–3.30)	2.55^**^ (0.92–4.18)
		Interaction term^a^				−0.87 (−2.69 to 0.96)

*CRCS, Children's Resilient Coping Scale; SDQ, Strength and Difficulties Questionnaire; 95%CI, 95% Confidence Interval*.

** *p < 0.01*,

* *p < 0.05*.

a *Interaction between leaving children at home alone and social capital*.

b *Adjusting for child sex, having a younger sibling, having an older sibling, living with grandparents, maternal age, marital status, maternal education, maternal occupation, income, K6, and social capital*.

c *Adding interaction term to Model 1*.

In terms of the SDQ, leaving children at home alone once a week or more was positively associated with the total difficulties score in crude model (β = 2.17, 95% CI = 0.79–3.55) and confounder-adjusted model (β = 2.22, 95% CI = 0.85–3.59) (Model 1). Because the point estimate did not attenuate toward null, potential confounders may not have confounded the association. However, conduct problems (β = 2.61, 95% CI = 0.62–4.60), hyperactivity/inattention (β = 3.38, 95% CI = 0.93–5.83), and peer relationship problems scores (β = 2.69, 95% CI = 0.89–4.49) showed substantial reduction of coefficients in leaving children at home alone once a week or more (Model 1). On the contrary, leaving children at home alone (both less than once a week and once a week or more) was not associated with emotional symptoms (β = 0.19, 95% CI = −1.83 to 2.20) and prosocial behavior scores (β = −1.36, 95% CI = −3.59 to 0.86). Interaction in terms of leaving children at home alone and social capital were not significant in all scores of the SDQ (Model 2). (all p for interaction term > 0.07), suggesting that high or low social capital did not differentiate the association between leaving children at home alone and SDQ score.

### Propensity-score matching

As shown in Table 1, characteristics were significantly different depending on the frequency of leaving children at home alone and can be considered to have confounded the association between leaving children at home alone and CRCS and SDQ. Thus, we conducted PS matching under two conditions (i.e., children who had never been left at home alone vs. children who had been left at home alone less than once a week, children who had never been left at home alone vs. children who had been left at home alone once a week or more). Table 3 shows the distribution of characteristics after PS matching. The covariate balance within the matched pairs was improved because the standardized bias of almost all covariates was less than 5%. Though some covariates had a standardized bias of more than 5%, the standardized bias of matched pairs decreased from that of the unmatched pairs or the results of their *t*-test were not significant.

**Table 3:** Distribution of characteristics after propensity-score matching.

		**Never**	** < 1/w**	**Bias (%)**	***p*-value**	**Never**	**≥1/w**	**Bias (%)**	***p*-value**
		**(*n* = 1,214)**	**(*n* = 1,214)**			**(*n* = 397)**	**(*n* = 397)**		
Child sex	Boy	610 (50.3)	624 (51.4)			198 (52.2)	202 (53.3)		
	Girl	602 (49.5)	589 (48.5)	−2.1	0.60	181 (47.8)	177 (46.7)	−2.1	0.77
	Missing	2 (0.2)	1 (0.1)	−2.4	0.56	0 (0)	0 (0)	0	NA
Younger sibling	Yes	548 (45.1)	540 (44.5)			121 (31.9)	126 (33.3)		
	No	666 (54.9)	674 (55.5)	−1.3	0.74	258 (68.1)	253 (66.7)	2.7	0.70
Older sibling	Yes	614 (50.6)	624 (51.4)			284 (74.9)	275 (72.6)		
	No	600 (49.4)	590 (48.6)	1.7	0.69	95 (25.1)	104 (27.4)	−5.3	0.46
Grandparents	Yes	105 (8.7)	126 (10.4)			27 (7.1)	26 (6.9)		
	No	1,109 (91.4)	1,088 (89.6)	5.6	0.15	253 (92.9)	353 (93.1)	−0.9	0.89
Maternal age	<30	64 (5.3)	63 (5.2)			22 (5.8)	26 (6.9)		
	30–34	240 (19.8)	223 (18.4)	−3.6	0.38	71 (18.7)	66 (17.4)	−3.4	0.64
	35–39	426 (35.1)	411 (33.9)	−2.6	0.52	130 (34.3)	127 (33.5)	−1.7	0.82
	40–44	349 (28.7)	387 (31.9)	−3.6	0.38	115 (30.3)	112 (29.5)	−1.7	0.81
	>45	166 (9.6)	110 (9.1)	6.7	0.09	34 (9.0)	39 (10.3)	4.3	0.54
	Missing	19 (1.6)	20 (1.6)	0.6	0.87	7 (1.9)	9 (2.4)	3.4	0.61
Marital states	Unmarried	74 (6.1)	100 (8.2)			47 (12.4)	51 (13.5)		
	Married	1,106 (91.1)	1,085 (89.4)	8.0	0.04	321 (84.7)	316 (83.4)	3.2	0.67
	Missing	34 (2.8)	29 (2.4)	−2.7	0.52	11 (2.9)	12 (3.2)	1.6	0.83
Maternal education	High school or less	407 (33.5)	454 (37.4)			155 (40.9)	149 (39.3)		
	Some college	533 (43.9)	493 (40.6)	−6.7	0.10	137 (36.2)	151 (39.8)	7.5	0.30
	College or more	249 (20.5)	240 (19.8)	−1.8	0.65	76 (20.1)	72 (19.0)	−2.6	0.71
	Other_unknown	25 (2.1)	27 (2.2)	1.1	0.78	11 (2.9)	7 (1.9)	−7.1	0.34
Maternal occupation	Full-time/Part-time	650 (53.5)	637 (52.5)			296 (78.1)	289 (76.2)		
	Self-employed	51 (4.2)	52 (4.3)	0.4	0.92	13 (3.4)	18 (4.7)	6.3	0.36
	Not-working/housewife	467 (38.5)	472 (38.9)	0.9	0.84	60 (15.8)	58 (15.3)	−1.3	0.84
	Missing	46 (3.8)	53 (4.4)	2.9	0.47	10 (2.6)	14 (3.7)	5.7	0.41
Income	<3 million yen	121 (10.0)	137 (11.3)			41 (10.8)	51 (13.5)		
	≥ 3 million yen	995 (82.0)	958 (78.9)	4.2	0.29	311 (82.1)	292 (77.0)	7.8	0.04
	Missing	98 (8.1)	119 (9.8)	6.0	0.14	27 (7.1)	36 (9.5)	8.0	0.34
K6	<5	870 (71.7)	876 (72.2)			256 (67.5)	245 (64.6)		
	≥5	342 (28.2)	336 (27.7)	−1.1	0.79	123 (32.5)	134 (35.4)	6.3	0.16
	Missing	2 (0.2)	2 (0.2)	0	1.00	0 (0)	0 (0)	0	NA
Social capital	Trust	654 (53.9)	629 (51.8)			187 (49.3)	201 (53.0)		
	Not trust	560 (46.1)	585 (48.2)	−4.1	0.31	192 (50.7)	178 (47.0)	7.4	0.31

*K6, Kesseler 6*.

Table 4 shows the coefficients of leaving children at home alone for CRCS and SDQ scores using fixed effect regression model. There was no significant association between leaving children at home alone less than once a week and never left children at home alone on both CRCS and SDQ scores. In contrast, leaving children at home alone once a week or more was positively associated with the total difficulties (β = 2.67, 95% CI = 0.76–4.59), conduct problems (β = 2.88, 95% CI = 0.14–5.61), hyperactivity/inattention (β = 3.46 95% CI = 0.02 to 6.91), and peer relationship problems scores (β = 3.38, 95% CI = 1.04–5.72) compared with children who had never been left at home alone. However, resilience (β = −2.10, 95% CI = −4.35 to 0.14), emotional symptoms (β = 0.98, 95% CI = −1.68 to 3.63), and prosocial behavior scores (β = −0.77, 95% CI = −3.78 to 2.25) were not significantly associated. These results remained unchanged when the participant was not the mother (data not shown).

**Table 4:** Coefficient of leaving children at home alone for the CRCS and the SDQ scores after propensity score matching.

		**Never**	**< 1/w**	**Never vs. < 1/w**	**Never**	**≥1/w**	**Never vs. ≥1/w**
		***n* = 1,214**	***n* = 1,214**	***n* = 2,428 (1,214 pairs)**	***n* = 379**	***n* = 379**	***n* = 758 (379 pairs)**
**Outcomes**		**Mean (*SD*)**	**Mean (*SD*)**	**β (95%CI)**	**Mean (*SD*)**	**Mean (*SD*)**	**β (95%CI)**
CRCS	Total	66.77 (15.36)	65.94 (15.24)	−0.83 (−2.04 to 0.38)	66.33 (15.42)	64.22 (15.88)	−2.10 (−4.35 to 0.14)
SDQ	Total difficulties score	24.59 (13.36)	24.63 (12.92)	0.04 (−0.98 to 1.06)	23.62 (12.83)	26.29 (13.43)	2.67^**^ (0.76–4.59)
	Emotional symptoms	19.81 (18.92)	18.97 (18.18)	−0.84 (−2.29 to 0.61)	18.68 (18.51)	19.66 (18.63)	0.98 (−1.68 to 3.63)
	Conduct problems	25.26 (19.14)	24.40 (17.61)	−0.86 (−2.33 to 0.62)	24.49 (18.31)	27.36 (19.45)	2.88^*^ (0.14–5.61)
	Hyperactivity/inattention	35.38 (23.18)	36.43 (22.98)	1.05 (−0.77 to 2.87)	34.62 (23.92)	38.07 (23.68)	3.46^*^ (0.02–6.91)
	Peer relationship problems	17.92 (16.83)	18.72 (16.53)	0.80 (−0.49 to 2.08)	16.70 (15.08)	20.08 (16.93)	3.38^**^ (1.04–5.72)
	Prosocial behavior	66.29 (20.40)	65.79 (19.49)	−0.50 (−2.08 to 1.08)	65.83 (20.43)	65.07 (21.10)	−0.77 (−3.78 to 2.25)

CRCS, Children's Resilient Coping Scale; SDQ, Strength and Difficulties Questionnaire; SD, Standard Deviation; 95% CI, 95% Confidence Interval

** p < 0.01,

* *p < 0.05*.

## Discussion

This is the first study to examine the association between leaving children at home alone and their mental health in a large cohort of Japanese children aged 6 or 7 years, focusing on both positive and negative aspects. Moreover, this study took into account the effect of social capital more than that of common confounders.

In terms of the association between leaving children at home alone and child mental health, the results indicated that children left at home alone frequently (i.e., once a week or more) was associated with only negative outcomes, specifically, the total difficulties, conduct problems, hyperactivity/inattention, and peer relationship problems scores, although children left at home alone occasionally (i.e., less than once a week) showed no impact on their mental health among 6 or 7 years children in Japan. The current findings are consistent with those of previous studies that revealed that leaving children at home alone increased the risk of accident or crime by children themselves (45), possibly because leaving children at home alone may escalate to further neglect toward children (7). Moreover, this study adds to the literature that there would be no impact of leaving children at home alone on the positive aspects of child mental health.

In this study, we found the impact of leaving children at home alone once a week or more on total difficulties, especially conduct problems, hyperactivity/inattention, and peer relationship problems. Previous studies showed that poor parent-child relationship is related to child conduct problems [e.g., (46)], hyperactivity/inattention [e.g., (47)], and peer relationship problems (48, 49). Therefore, leaving children at home alone might be associated with parent-child relationships. Further study is needed to examine the mediating role of both community environment and parent-child relationship. Moreover, conduct problems and hyperactivity/inattention are often classified as “externalizing” problems (50). In terms of peer relationship, poor peer relationship predicts externalizing behavior and internalizing behaviors (51, 52). Hence, it is likely to be more difficult for parents to detect children internalizing problems (i.e., emotional problems) than externalizing problems (46), which might be caused by children's need for attention from the parents.

To interpret the current findings, it might be helpful to understand the cultural difference between the Japanese and Western cultures, which are characterized by interdependence and independence, respectively (13). Interdependence is a social orientation that is more likely to emphasize on harmony or relatedness, which is more likely found in Japan. Independence is a social orientation that is more likely to emphasize on self-direction, autonomy, and self-expression, which can be observed more in the Western culture. These differences may affect the parenting style in these cultures; Japanese parents are less likely to encourage autonomy and children's personal choice (53), while American parents are more likely to emphasize on individualism or autonomy [e.g., (54)]. Additionally, Japanese parents might expect interdependence with the community, which can be related with social capital (55). Our findings indicated that the negative impact of leaving children at home alone on child mental health remained even though we took into account of the effect of social capital. That is, we showed the adverse effect of leaving children at home alone in Japan, where autonomy is less likely focused in parenting, and the level of interdependence in the community, measured as the social capital, had no impact on this association.

There are several limitations to this study. First, a causal relationship between leaving children at home alone and child mental health cannot be revealed because it was a cross-sectional study. A previous study suggested that the parent–child relationship might deteriorate if the child is left at home alone (6). It is necessary to examine the long-term effects of leaving children at home alone through a longitudinal study. Second, all variables in this study were assessed only by the parent or caregiver. Ideally, the SDQ and resilience should be assessed not only by caregivers, but also by school teachers. Further, the absence of the responses of children themselves might induce a measurement error. Moreover, there are some limitations in accurately detecting the variables related to child mental health using only parental assessment. However, we should be cautious when using the self-rating SDQ with children younger than 11 years (56). It is necessary to understand child behavior as accurately as possible using the teacher-rated SDQ. Third, the participants of this study were children in Adachi City, Tokyo, which is an urban area in Japan. The association between leaving children at home alone and child mental health may differ depending on regional characteristics, public safety, culture, including concept of values, and so on. Especially, these results might not be applicable to other areas such as rural towns. However, child poverty is expected to become a more serious problem in Japan (57). We need to examine the impact of leaving young children at home alone in Adachi City continually and in other areas. Fourth, PS matching may increase the imbalance of covariates and may lead to a selection bias (58). In this study, we checked the imbalance and confirmed that it may not have affected the result of the study.

In conclusion, we found that leaving children at home alone may be linked to child conduct problems or hyperactivity problems, and it was not associated with other positive aspects of mental health. Leaving children at home alone should not be recommended in Japan, similar to the recommendation in North America.

## Author contributions

TF conceived the study, TF, MO, AI and TK managed study and collected data, SD wrote first draft, TF finalized the manuscript. All authors have read and approved the final manuscript.

### Conflict of interest statement

The authors declare that the research was conducted in the absence of any commercial or financial relationships that could be construed as a potential conflict of interest.

## Footnotes

^1^State of Maryland Family Law 5-801. Confinement in dwelling, building, enclosure, or motor vehicle. Provided by Anne Arundel County Department of Social Services, Annapolis, Maryland. Contact: Madelyn Williams, Protective Supervisor, [410] 269-4701
